# TIR domain proteins: regulatory mechanisms in the tumor immune microenvironment, clinical translation strategies, and prospects for precision therapy applications

**DOI:** 10.3389/fimmu.2025.1695754

**Published:** 2025-12-18

**Authors:** Jiatian Lou, Chenlei Gong, Xiaotao Gao, Jiaren Zhou, Qiyuan Wu, Xiaoliang Zheng, Liyan Cheng

**Affiliations:** 1 Clinical Medical College, Hangzhou Medical College, Hangzhou, Zhejiang, China; 2 School of Laboratory Medicine and Bioengineering, Hangzhou Medical College, Hangzhou, Zhejiang, China; 3 Zhejiang Key Laboratory of Tumor Molecular Diagnosis and Individualized Medicine, Hangzhou Medical College, Hangzhou, Zhejiang, China

**Keywords:** Toll/IL-1R domain proteins, tumor immune microenvironment, clinical translation, cancer, immunotherapy

## Abstract

Toll/IL-1R (TIR) domain proteins, as central signaling hubs in innate immunity, dynamically orchestrate inflammatory responses and immune processes within the tumor microenvironment (TME) by mediating both MyD88-dependent and TRIF-dependent pathways. This review systematically elaborates on the dual regulatory roles of the TIR superfamily-encompassing toll-like receptors (TLRs), IL-1 receptors (IL-1Rs), and adaptor proteins-in tumor immunity, including the facilitation of stemness maintenance in cancer stem cells (CSCs) and the inductive mechanisms driving the formation of an immunosuppressive TME. From the perspective of clinical translation, the combinatorial therapeutic strategy of TIR agonists/inhibitors with immune checkpoint inhibitors (ICIs) represents a novel paradigm: the synergistic effects among TIR agonists/inhibitors, advanced nanodelivery systems, and radiotherapy-responsive prodrug technology provide a potential approach to address challenges such as systemic toxicity and low targeted delivery efficiency. Looking forward, the continuous advancement and broader application of TIR protein targets in the field of precision cancer immunotherapy hold great promise for offering new hope in the fight against malignant tumors.

## 1 Introduction

The immune system is structurally divided into two components: innate immunity (also known as natural or non-specific immunity) and adaptive immunity. In vertebrates, the adaptive immune response is a relatively slow process. The innate immune system serves as the first line of defense against infectious pathogens and endogenous danger signals such as cancer. It functions by recognizing conserved molecular structures of pathogens—known as pathogen-associated molecular patterns (PAMPs)—as well as endogenous ligands released by damaged cells, referred to as damage-associated molecular patterns (DAMPs), and mounting responses accordingly. Innate immunity is evolutionarily conserved and exists in nearly all multicellular organisms (1). During innate immune responses, pattern recognition receptors (PRRs), such as membrane-bound TLRs, RIG-I-like receptors (RLRs), C-type lectin receptors (CLRs), and NOD-like receptors (NLRs), are responsible for recognizing PAMPs/DAMPs. Upon recognition, these PRRs recruit specific adaptor proteins in distinct cellular compartments, thereby initiating multiple downstream signaling cascades (2–4). TLRs, as key members of the PRR family, all contain a highly conserved TIR domain in their intracellular region, which plays a central role in signal transduction. Upon ligand recognition, the TIR domain recruits adaptor proteins such as MyD88 and TRIF, thereby activating downstream NF-κB and interferon regulatory factor (IRF) signaling pathways. This ultimately induces the production of pro-inflammatory cytokines and type I interferons, initiating an anti-tumor immune response (5–7). In addition, recent studies have revealed that the function of the TIR domain extends well beyond serving as a simple protein-protein interaction scaffold. It is widely distributed in both prokaryotes and eukaryotes and exhibits enzymatic activity as an NAD+(nicotinamide adenine dinucleotide)-degrading enzyme (8, 9).

The TME is not an isolated system per se, but rather the result of dynamic interplay among diverse cellular and non-cellular components. It constitutes a dynamic system encompassing immune cells, cytokine networks, stromal cells (such as cancer-associated fibroblasts (CAFs) and endothelial cells), tumor cells, and the extracellular matrix (ECM). An immunosuppressive TME represents a complex ecosystem that enables tumor survival and progression, characterized by exhausted effector T cells, expansion of regulatory T cells (Tregs), accumulation of myeloid-derived suppressor cells (MDSCs), and sustained expression of numerous immunosuppressive molecules. The establishment of this immunosuppressive milieu depends not only on intrinsic features of tumor cells, but is also closely linked to an active immune escape network shaped by the tumor through multiple signaling pathways (10, 11). Within this network, TIR domain proteins, as evolutionarily conserved immune signaling hubs, play a notable role in fostering the immunosuppressive TME (12, 13).

Given the central role of TIR domain proteins in innate immunity and the tumor microenvironment, this study aims to systematically review the signaling mechanisms of TIR domain proteins, elucidating how TIR-mediated pathways influence both the biological behavior of tumor cells and the activation, differentiation, and functional states of tumor-infiltrating immune cells. Furthermore, it provides an in-depth discussion of recent advances in TIR-targeted tumor immunotherapy strategies, including preclinical and clinical research progress, current challenges, and future directions. The ultimate goal is to evaluate the translational potential of these strategies and to establish a solid theoretical foundation and innovative perspectives for developing more effective cancer immunotherapies.

## 2 Overview of TIR domain proteins

### 2.1 The TIR superfamily

**Figure 1:**
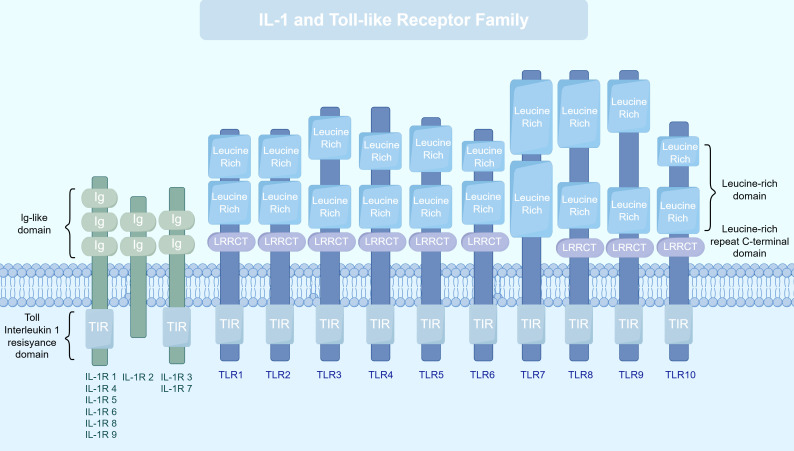
Comparison of structural features between the TLR and IL-1R protein families. On the left is the IL-1Rs family, which belongs to the immunoglobulin superfamily. Their extracellular regions contain two or three immunoglobulin-like domains responsible for binding corresponding cytokines. Their intracellular regions all contain a conserved TIR domain. A special case is IL-1R2, which lacks the intracellular TIR domain and functions as a decoy receptor. It negatively regulates signaling by “trapping” ligands or the co-receptor (IL-1RAcP). On the right are the TLRs, which are Type I transmembrane proteins. Their extracellular regions typically consist of an N-terminal cap (LRRNT), a series of leucine-rich repeats (LRRs), and a C-terminal cap (LRRCT), and are responsible for recognizing various PAMPs or DAMPs. Notably, the extracellular region of TLR7 lacks the typical LRRCT motif; its C-terminus is replaced by a unique insertion loop, a structural feature closely related to its specific recognition of nucleic acid ligands. The transmembrane domain anchors the receptor in the plasma membrane or endosomal membrane. The intracellular region invariably contains a conserved TIR domain, which initiates downstream signal transduction. This figure clearly illustrates the fundamental structural distinctions between these two major receptor superfamilies, as well as the conservation of their signal initiation module (the TIR domain).

TIR domain proteins constitute a highly evolutionarily conserved superfamily, widely distributed across diverse organisms from bacteria to humans, where they play indispensable roles in innate immune defense and inflammatory responses (2). A defining feature of this superfamily is the presence of an intracellular TIR domain, approximately 200 amino acids in length, which adopts a distinct spatial conformation that mediates protein-protein interactions and signal transduction (14). A detailed comparison of the members in the TIR superfamily are as shown in (**Figure 1**). Notably, the TIR domain also serves essential functions in the immune systems of plants and bacteria, underscoring its cross-species conservation in immune defense mechanisms (15, 16).

From the perspective of molecular evolution, TIR domain proteins likely originated in prokaryote (8), and were subsequently diversified in eukaryotes through both vertical inheritance and horizontal gene transfer, giving rise to a variety of functional adaptations (17). In mammals, TIR domains are primarily found in three classes of proteins: TLRs, IL-1R family, and TIR domain-containing adapter proteins (such as MyD88) (18). Although these proteins recognize distinct ligands and participate in different physiological processes, they all initiate downstream signaling through the conserved TIR domain, illustrating a unity of mechanistic conservation amid functional diversity (19). Structurally, all TIR domains share three highly conserved sequence motifs (Box 1, Box 2, and Box 3), which collectively form the structural framework for the three-dimensional folding of the domain (20).

### 2.2 The TLR subfamily

The TLR subfamily represents one of the most important classes of pattern recognition receptors within the TIR superfamily, forming the first line of defense against pathogenic invasion (21). TLRs are type I transmembrane proteins characterized by an extracellular leucine-rich repeat (LRR) domain, a single transmembrane region, and an intracellular TIR domain. In humans, 10 functional TLRs (TLR1–TLR10) have been identified, whereas mice possess 12 (TLR1–TLR13). These receptors can be categorized into two major groups based on their subcellular localization and ligand specificity: cell surface TLRs and intracellular TLRs. The Key features and functions of human TLRs are as shown in (**Table 1**).

**Table 1:** Key features and functions of human toll-like receptors (TLRs).

TLR	Localization	Dimerization form	Recognized ligand(s)	Adaptor protein(s)	Primary response
TLR1 (237)	Cell Membrane	Heterodimer with TLR2	Triacylated lipopeptides	MyD88/MAL	Pro-inflammatory cytokines
TLR2 (238)	Cell Membrane	Homodimer or heterodimer with TLR1/6	Various lipoproteins	MyD88/MAL	Pro-inflammatory cytokines
TLR5 (239)	Cell Membrane	Homodimer	Flagellin	MyD88	Pro-inflammatory cytokines
TLR6 (238)	Cell Membrane	Heterodimer with TLR2	Diacylated lipopeptides	MyD88/MAL	Pro-inflammatory cytokines
TLR10 (240)	Cell Membrane	Homodimer	Unknown	MyD88	Unknown
TLR3 (239)	Endosomal Membrane	Homodimer	Double-stranded RNA	TRIF	Type I interferons
TLR7 (241)	Endosomal Membrane	Homodimer	Single-stranded RNA	MyD88	Type I interferons
TLR8 (241)	Endosomal Membrane	Homodimer	Single-stranded RNA	MyD88	Type I interferons
TLR9 (241)	Endosomal Membrane	Homodimer	CpG DNA	MyD88	Type I interferons
TLR4 (238)	Cell Membrane/ Endosomal Membrane	Homodimer	LPS, HSP, etc.	MyD88/MAL/ TRIF/TRAM	Pro-inflammatory cytokines/Type I interferons

The ligand recognition mechanisms of TLRs exhibit high specificity and diversity. Cell surface-localized TLRs (e.g., TLR1, TLR2, TLR4, TLR5, TLR6, TLR10) primarily recognize membrane components of microbes such as bacteria and fungi (22). For instance, TLR4 identifies lipopolysaccharide (LPS) from Gram-negative bacteria (23, 24), while TLR2 recognizes bacterial lipopeptides by forming heterodimers with either TLR1 or TLR6 (25). In contrast, TLRs located on endosomal membranes (e.g., TLR3, TLR7, TLR8, TLR9) mainly target nucleic acid ligands: TLR3 detects viral double-stranded RNA, TLR7/8 recognize viral single-stranded RNA, and TLR9 binds to CpG motifs in bacterial DNA (26). This compartmentalized recognition strategy enables TLRs to comprehensively surveil both extracellular and intracellular PAMPs (27).

The activation of TLRs relies on signal transduction mediated by their TIR domains. Upon recognition and binding of specific ligands, conformational changes occur in the extracellular domain of TLRs, leading to exposure of the intracellular TIR domain and facilitating the formation of homodimers or heterodimers. This dimerization creates a platform for the recruitment of downstream adaptor proteins. TLR signaling is primarily transmitted through two major pathways: the MyD88-dependent pathway and the TRIF-dependent pathway (28, 29). With the exception of TLR3, all TLRs utilize the MyD88-dependent pathway. In contrast, TLR3 and TLR4 can activate the TRIF-dependent pathway, with TLR4 being the only receptor capable of initiating both pathways (24).

TLRs are closely associated with various human diseases, including autoimmune disorders, infectious diseases, and cancer. For example, aberrant activation of TLR7 and TLR9 has been linked to the pathogenesis of systemic lupus erythematosus (SLE) (30). Polymorphisms in the TLR3 gene are significantly associated with an increased risk of type 1 diabetes mellitus (31) and several cognitive behavioral disorders (32, 33). Mutations in TLR4 are correlated with susceptibility to Gram-negative bacterial infections, and TLR4 deficiency has been shown to alleviate LPS-induced acute liver injury by suppressing the TLR4/MyD88/NF-κB signaling pathway (23). Within the TME, TLR activation can either promote anti-tumor immunity or facilitate tumor progression through chronic inflammation. This dual role is highly context-dependent, varying according to the receptor subtype and tumor type. These findings position TLRs as promising targets for immunotherapy and vaccine development (34). The following sections will focus specifically on their applications in cancer immunotherapy.

### 2.3 The IL-1R subfamily

The IL-1R subfamily represents another major branch of the TIR superfamily, primarily mediating inflammatory responses and immune regulation (18). Unlike TLRs, IL-1R members recognize endogenous cytokines rather than pathogen-derived molecules, serving as a critical bridge linking innate and adaptive immunity. Belonging to the immunoglobulin superfamily (IgSF), IL-1R members contain immunoglobulin-like domains in their extracellular region and a canonical TIR domain intracellularly, activating downstream inflammatory pathways through similar signal transduction mechanisms (35).

The IL-1R family includes several members, which can be categorized based on function and structure as follows: Type I IL-1 receptor (IL-1R1) is the primary signaling receptor for IL-1α and IL-1β (36); Type II IL-1 receptor (IL-1R2) acts as a “decoy receptor”—lacking an intracellular TIR domain, it binds IL-1 but does not initiate signaling (37); IL-1 receptor accessory protein (IL-1RAcP) serves as a co-receptor for IL-1R1 and is essential for signal transduction (38); ST2 (IL-1R4) is the specific receptor for IL-33 and participates in Th2 immune responses (39); IL-18Rα and IL-18Rβ together form the IL-18 receptor complex; and IL-1Rrp2 (IL-1R6) functions as an auxiliary receptor for the IL-36 receptor (40).

The signaling activation mechanism of the IL-1R family exhibits high structural specificity. Taking classical IL-1 signaling as an example, IL-1β binding to IL-1R1 induces conformational changes in the receptor and recruits IL-1RAcP to form a heterotrimeric complex (36, 41). This assembly brings two TIR domains into spatial proximity, providing a platform for the binding of the downstream adaptor protein MyD88. Subsequently, MyD88 binds to the receptor complex via its TIR domain and further recruits IRAK family kinases, activating NF-κB and MAPK signaling pathways similar to those in TLR signaling. This ultimately leads to the expression of various pro-inflammatory cytokines, such as TNF-α and IL-6 (14).

The IL-1 signaling system plays a crucial role in maintaining immune homeostasis, and its activity is tightly regulated at multiple levels to prevent excessive inflammatory responses that could harm the host. Regulatory mechanisms include competitive inhibition of IL-1 receptors by the IL-1 receptor antagonist (IL-1Ra), the decoy function of IL-1R2, negative regulation by soluble IL-1RAcP, and involvement of inhibitory molecules such as SIGIRR. Dysregulation of these mechanisms is closely associated with the development of various autoinflammatory diseases, underscoring the importance of precise control in maintaining immune equilibrium (38, 39). From a molecular structural perspective, although the TIR domains of IL-1R members share similar signal transduction mechanisms with TLRs, they exhibit notable functional differences. Unlike TLRs, the TIR domains of IL-1Rs do not contain a death domain and primarily transduce signals through activation of NF-κB and MAPK pathways (42). Moreover, IL-1R family members often require auxiliary receptors (e.g., IL-1RAcP) to form functional signaling complexes, contrasting with the homodimeric or heterodimeric activation mechanisms typical of TLRs. These structural and mechanistic differences reflect functional diversification during the evolution of TIR domain proteins and provide important insights into the specific roles of various immune receptors in inflammatory responses (14, 43).

The tissue distribution and expression regulation of IL-1R family members also hold significant physiological implications. IL-1R1 is widely expressed in various cell types, including immune cells, epithelial cells, and endothelial cells, whereas IL-1R2 is primarily expressed on B cells, monocytes, and neutrophils. ST2 (IL-33R) is highly expressed on Th2 cells, mast cells, and regulatory T cells. These distinct expression patterns determine the tissue-specific responses and functional diversity toward IL-1 family cytokines (35).

The IL-1R family plays a key role in various pathological processes. Within the tumor microenvironment, IL-1 influences cancer progression through multiple mechanisms, including induction of DNA damage molecules, stimulation of angiogenesis, and modulation of immune cell activity (43, 44). Clinical studies have shown that elevated IL-1 expression is often associated with poor patient prognosis, while blockade of IL-1 signaling can effectively inhibit tumor metastasis (45). Based on these findings, the IL-1R signaling pathway has been identified as an important molecular target for the treatment of various malignancies.

### 2.4 TIR domain-containing adapter proteins

TIR domain-containing adapter proteins are key molecules within the TIR superfamily that do not directly participate in ligand recognition but are specialized in signal transduction, serving as bridges connecting receptors with downstream effector molecules. These proteins share the common feature of containing a TIR domain while lacking transmembrane and extracellular domains. Major members include MyD88, TIRAP/Mal, TRIF, and TRIF-related adapter molecule (TRAM) (46–48). Through their TIR domains, these adapter proteins interact with receptors to form specific signal transduction complexes, determining the specificity and efficiency of downstream pathways.

MyD88 is the central adapter protein in both TLR and IL-1R signaling pathways. Nearly all TLRs (except TLR3) depend on MyD88 for signal propagation. Structurally, MyD88 comprises three functional domains: a C-terminal TIR domain responsible for interacting with receptor TIR domains; a middle linker region; and an N-terminal death domain (DD) that recruits downstream kinases (49). Upon receptor activation, MyD88 is recruited to the receptor complex via TIR–TIR interactions. It then recruits IRAK family kinases (IRAK4 and IRAK1/2) through its death domain, forming a supramolecular complex known as the “myddosome” (50, 51). The assembly of this multi-protein complex triggers subsequent signaling cascades, ultimately leading to the activation of NF-κB and MAPK pathways and the production of pro-inflammatory cytokines (52).

TIRAP/Mal is a critical bridging protein in the TLR2 and TLR4 signaling pathways, specifically responsible for recruiting MyD88 to receptor complexes at the plasma membrane (53). Unlike MyD88, Mal does not possess a death domain and therefore cannot directly activate downstream signaling molecules. The TIR domain of Mal has unique structural features: it contains a long AB loop but lacks the BB loop commonly found in other TIR domains. Crystallographic studies have shown that Mal forms dimers in a two-fold symmetric manner, with its AB loop region simultaneously binding both TLR4 and MyD88, thereby spatially bridging these two proteins. Mutations in key residues of the AB loop or the dimer interface of Mal significantly impair TLR4 signaling, underscoring the functional importance of this structural feature.

TRIF (TIR domain-containing adapter-inducing interferon-β) is the central adapter protein in the MyD88-independent pathway, primarily recruited by TLR3 and TLR4. In contrast to MyD88, TRIF activates two parallel downstream pathways: one activates NF-κB through RIP1 kinase, and the other activates interferon regulatory factor 3 (IRF3) via TANK-binding kinase 1 (TBK1) and IKKϵ, leading to the production of type I interferons. This dual-pathway activation mechanism enables TLR3 and TLR4 to simultaneously trigger inflammatory responses and antiviral immunity, reflecting the multifunctionality of the innate immune system. The activity of TRIF is further modulated by TRAM (TRIF-related adapter molecule), which specifically assists TLR4 (but not TLR3) in recruiting TRIF, adding another layer of complexity to the signaling network. A seminal study reveals that the TIR domains of human TRIF and TRAM spontaneously form two-stranded, parallel helical filaments. This assembly is stabilized by two critical interfaces: an intrastrand “BE” interface, mediated by the canonical BB loop, and an interstrand “BCD” interface. This architectural blueprint is strikingly similar to the filamentous assemblies of MyD88 and MAL, proposing a unified and evolutionarily conserved “cooperative assembly formation” mechanism for signal transduction across all major TLR adaptors. Mutagenesis studies confirm that disrupting these interfaces abrogates TLR4/TRIF-mediated NF-κB activation. This model elegantly demonstrates how activated TLRs nucleate the helical polymerization of adaptor TIR domains to initiate and amplify downstream signaling (48).

### 2.5 The structure–function relationship of the TIR domain

The three-dimensional structural features of the TIR domain are closely linked to its function, a relationship that has been highly conserved throughout evolution. The core of the TIR domain consists of an α/β fold composed of approximately 200 amino acids, featuring a central parallel β-sheet (typically formed by five β-strands) surrounded by several α-helices. Structural biology studies have revealed that the functional specificity of TIR domains is primarily determined by the spatial conformation of their surface loop regions and the three-dimensional distribution of conserved amino acid residues (49). Although sequence homology among TIR domains from different origins may be as low as 20–30%, their spatial architectures exhibit remarkable similarity, reflecting extreme functional conservation throughout evolution.

Characteristic structural elements of the TIR domain include three highly conserved sequence motifs (Box 1, Box 2, and Box 3) and several key loop regions. Box 1 (F/YDAFISY) is located in the N-terminal region and contains a short α-helix (αA) and part of a β-sheet (βA), contributing to the core stability of the TIR domain. Box 2 (GYKLC-RD-PG) includes a surface-exposed “BB loop” (situated between the αB helix and the βB strand), which serves as a critical region mediating protein–protein interactions. Box 3 contains a conserved tryptophan residue surrounded by basic amino acids and is essential for maintaining structural integrity (54). Studies indicate that functional TIR–TIR interactions typically involve two symmetric interfaces: one primarily formed by the Box 1 and Box 2 regions, and the other by Box 3 and adjacent regions. This dual-interface interaction mode ensures the specificity and stability of signal complex assembly (20). Furthermore, Box 1 and Box 3 maintain their structural conformation even under high temperatures, whereas the dynamic flexibility of the BB loop in Box 2 may influence signaling efficiency (55).

The BB loop is one of the most characteristic functional elements in the TIR domain and is highly conserved across most TLRs (except TLR3) and IL-1Rs. This loop region is rich in positively charged arginine residues, forming an electrostatic “charge patch” that facilitates interactions with the TIR domains of downstream adapter proteins, such as MyD88 (56). The conformation and length of the BB loop vary among different TIR domains, and this variability is associated with the specificity of signal transduction regulation. For instance, as demonstrated by the Clabbers group, TLR4 utilizes the unique structure of Mal to act as a molecular bridge, simultaneously engaging both TLR4 and MyD88. This mechanism may also explain why TLR4 possesses a longer BB loop, providing a larger interaction interface to coordinate the recruitment of dual adapters. These structural features clarify why TLR4 can recruit a broader variety of adapter proteins (e.g., TRAM), while other receptors such as TLR2 primarily rely on TIRAP. TLR3 represents an exception, as its BB loop region is replaced by three conserved alanine residues, which may account for its inability to recruit MyD88 and its exclusive use of the TRIF-dependent pathway (32).

**Figure 2:**
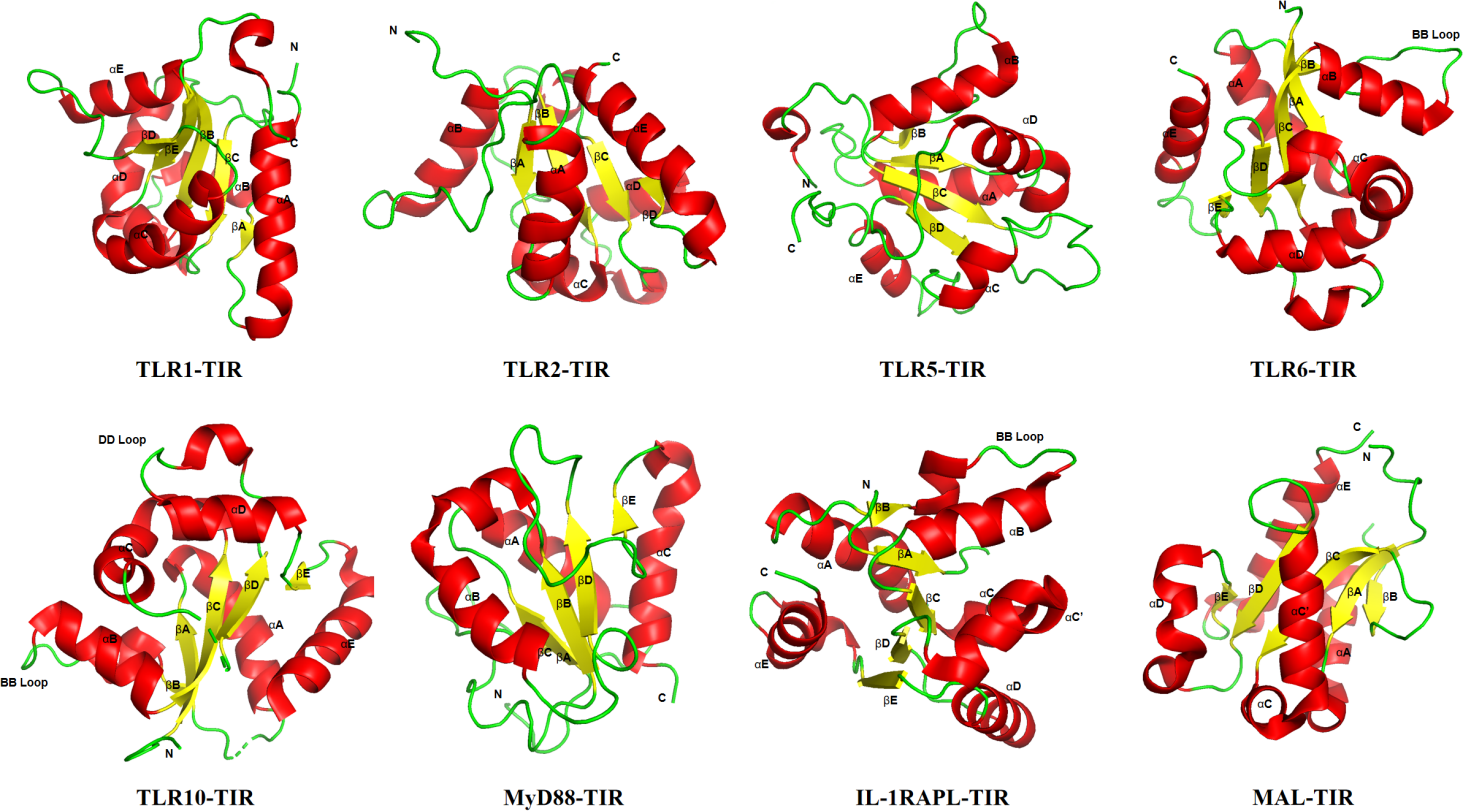
Architectural overview of the TIR domain in the TLR/IL-1R superfamily.

The function of TIR domains is closely linked to their oligomerization state. During signal transduction, multiple TIR domains form homo- or heterodimers mediated through the BB loop. For example, the TIR domain of MyD88 forms a head-to-tail dimer conformation facilitated by the BB loop, a structural feature that promotes the exposure of the death domain (DD) and the subsequent recruitment of IRAK kinases. In TLR4 signaling, dimerization of its TIR domain plays a central role. Studies have revealed diverse oligomerization patterns among TIR domains: Mal/TIRAP forms dimers via two-fold symmetry, whereas TRIF may assemble into more complex oligomeric structures. An overview of the structure of the TIR domain in the TLR/IL-1R superfamily is shown in the (**Figure 2**), and the key structural features and functional associations of the domains are shown in the (**Table 2**).

**Table 2:** Key structural features and functional correlations of the TIR domain.

Structural feature	Location	Consensus sequence	Functional role	Associated mutations and diseases
Box1 (242)	N-terminal region	F/YDAFISY	Maintains core structural stability	TLR5 mutation → unresponsiveness to flagellin
Box2 (BB loop) (243)	Between αB and βB	PG-X(4-7)-WP	Mediates TIR–TIR interactions	TLR4 P714H → endotoxin tolerance
Box3 (242)	Near C-terminus	Conserved Trp	Participates in signal complex assembly	MyD88 L93P → primary immunodeficiency
AB loop	Variable region	Highly variable	Specific protein recognition	MAL AB loop mutation → signaling deficiency
αC Helix	Central region	Hydrophobic core	Maintains structural integrity	IRAK4 V81I → susceptibility to pyogenic infections

Functional regulation of TIR domains is also closely tied to their structural dynamics. These domains are not static rigid molecules; their loop regions exhibit significant conformational plasticity (57). This flexibility allows TIR domains to adaptively adjust according to the requirements of different binding partners, enabling multi-specific recognition. A notable example is the interaction between TLR4 and TIRAP, during which the TIR domain of TLR4 undergoes a substantial conformational change—the BB loop transitions from a closed to an open state, exposing key interaction sites (58). This conformational switch is critical for the precise assembly of the signaling complex.

Although the three-dimensional structure of the TIR domain has been preliminarily resolved, how its conformational dynamics in different receptor contexts precisely regulate signaling specificity remains a challenging research focus. For instance, although TLR3 and TLR4 both utilize the TRIF pathway, their signal output intensity and duration differ significantly (59). Whether this divergence is linked to the conformational flexibility of their TIR domains awaits further clarification through structural biology studies.

## 3 Immune signaling pathway mediated by TIR domain protein

### 3.1 MyD88-dependent pathway

The MyD88-dependent pathway serves as the primary route for TLR signal transduction, with the vast majority of TLRs—except TLR3—relying on this pathway for signal transmission (60, 61). TLR1–6 heterodimers preferentially recognize membrane components of pathogens on the cell surface, while TLR7–9 detect nucleic acids derived from both host and foreign microorganisms (62). Upon ligand recognition, TLRs recruit the adapter protein MyD88, which contains a homologous TIR domain, via their intracellular TIR domains (63). The C-terminal TIR domain of MyD88 interacts with the TIR domain of the TLR (47, 64), while its N-terminal DD further recruits members of the IRAK family, such as IRAK4, IRAK1, IRAK2, and IRAK-M, forming the Myddosome signaling complex (65). The assembly of this complex triggers a series of phosphorylation cascades (66), involving IRAK1, the E3 ubiquitin ligase TRAF6, and K63-linked polyubiquitin chains, leading to the activation of TAK1. Activated TAK1 subsequently phosphorylates and activates the canonical IKK complex (67), ultimately resulting in the activation of key transcription factors such as NF-κB and AP-1. This process induces the production of pro-inflammatory cytokines including TNF-α, IL-6, and IL-12 (68, 69).

In the context of the tumor microenvironment, the MyD88-dependent pathway exhibits a dual role in immune function. On one hand, moderate activation of this pathway can promote anti-tumor immune responses. Activation of MyD88 signaling in myeloid cells enhances dendritic cell maturation and antigen presentation capacity (70), thereby facilitating the activation and proliferation of CD8^+^ T cells (71). On the other hand, sustained and excessive activation of MyD88 signaling contributes to the formation of an immunosuppressive microenvironment (72). In various solid tumors, such as ovarian (73, 74), colorectal (75, 76), and pancreatic cancers (77, 78), aberrant activation of the MyD88 pathway in tumor-infiltrating myeloid cells leads to excessive production of immunosuppressive cytokines like IL-10 and TGF-β. These cytokines directly suppress the function of effector T cells and promote the expansion and recruitment of Tregs (79).

### 3.2 TRIF-dependent pathway

**Figure 3:**
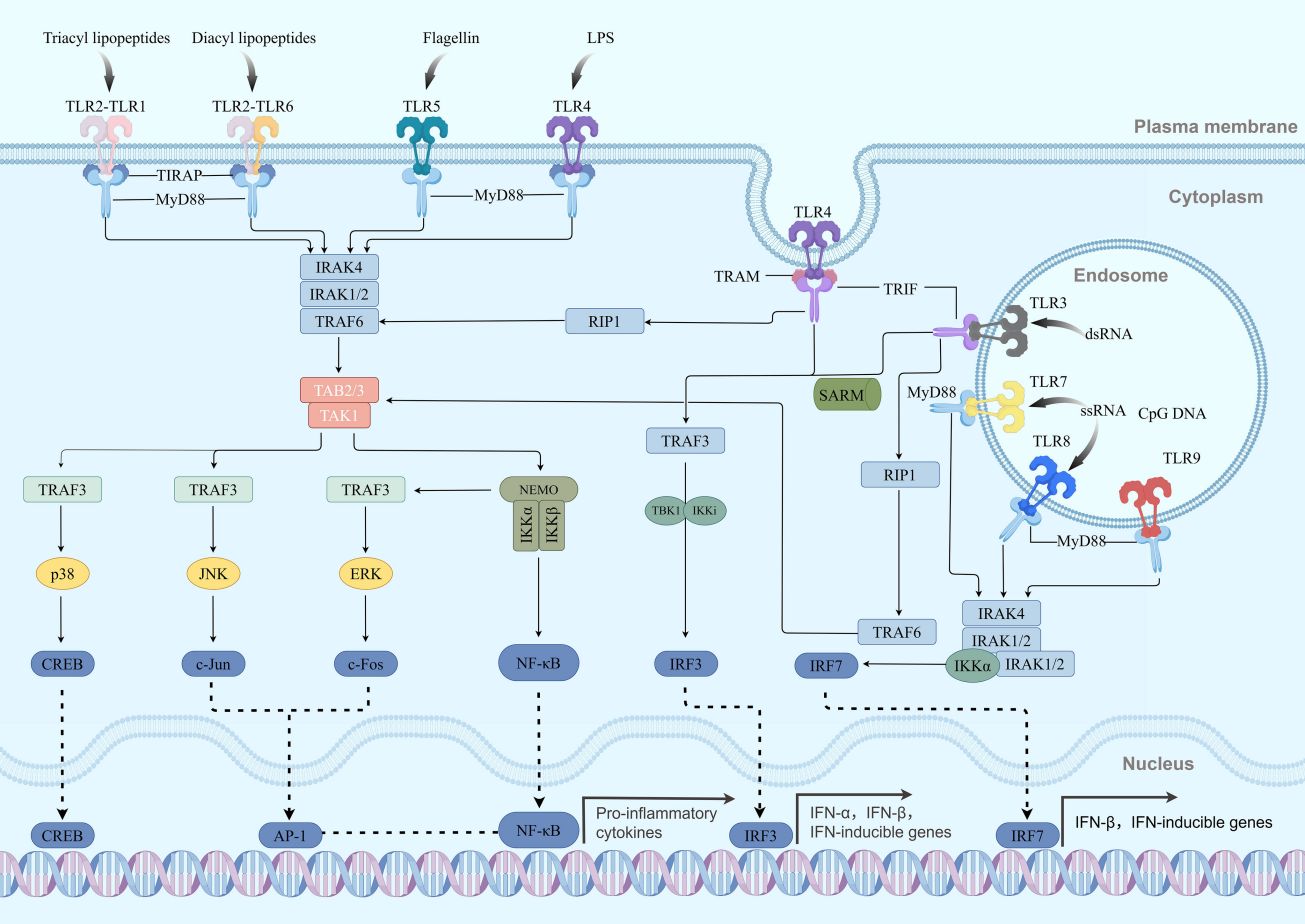
Panorama of TIR domain protein-mediated MyD88-dependent and TRIF-dependent signaling pathways. Upon recognition of their respective ligands by cell surface TLRs, including TLR4, TLR2/1 or TLR2/6 heterodimers, and TLR5, these receptors recruit MyD88 via TIRAP. Most TLRs, except for TLR3, can also recruit MyD88 directly. MyD88, in turn, recruits IRAK4 and IRAK1/2 through its DD, forming the myddosome complex. Activated IRAK1 then promotes TRAF6 to synthesize K63-linked polyubiquitin chains, leading to the activation of the TAK1 complex. Subsequently, TAK1 phosphorylates and activates both the IKK complex (resulting in NF-κB activation) and the MAPK pathways (activating JNK, p38, ERK). These events coordinately induce transcription factors such as AP-1, ultimately driving the production of pro-inflammatory cytokines. On the other hand, endosomally localized TLR3, which recognizes dsRNA, directly recruits TRIF. TLR4, upon internalization after activation, recruits TRIF via TRAM. TRIF then propagates the signal through two branches: one branch involves the recruitment of TRAF6 and RIP1, leading to the activation of TAK1 and subsequently NF-κB/MAPK pathways; the other branch involves the recruitment of TRAF3, which activates the TBK1/IKKϵ complex. This complex phosphorylates IRF3, prompting its dimerization and nuclear translocation, thereby inducing the production of type I interferons (IFN-α/β). Notably, in plasmacytoid dendritic cells (pDCs), TLR7/8/9 signaling can also directly activate IRF7 via IRAK1 and IKKϵ, potently inducing type I interferon production.

TRIF is a key adaptor protein in the MyD88-independent pathway, primarily involved in the signal transduction of TLR3 and TLR4 (29, 80, 81). Among these, TLR4 participates in the MyD88 pathway on the membrane, and upon activation, it is endocytosed into endosomes, thereby switching its signaling to the TRIF pathway (62). Unlike the MyD88 pathway, the TRIF pathway activates immune responses through the following mechanism: when TLR3 recognizes its ligand (such as double-stranded RNA) or TLR4 recognizes LPS, it recruits TRIF via the TRAM adaptor protein, forming the TRIF signaling complex (82). TRIF subsequently recruits TBK1 and RIP1 (83), while also activating IKK, thereby activating IRF3 (84) and NF-κB (85). The activated IRF3 translocates to the nucleus and induces the production of type I interferons (86, 87). This pathway is critical in antiviral and antitumor immunity but can also be hijacked by tumor cells to promote immune escape (88). Immune signaling pathways mediated by TIR domain proteins are shown in the (**Figure 3**). MyD88-dependent versus TRIF-dependent pathway differences are shown in the (**Table 3**).

**Table 3:** Key differences between MyD88-dependent and TRIF-dependent pathways.

Characteristic	Major receptor	Major adaptor protein	Major transcription factor	Major effector molecule
MyD88-Dependent Pathway	TLR2, TLR4-9 (except TLR3)	MyD88, IRAK1/4	NF-κB, AP-1	TNF-α, IL-6, IL-12 and other pro-inflammatory factors
TRIF-Dependent Pathway	TLR3, TLR4	TRIF, TRAM, TBK1	IRF3, NF-κB (delayed activation)	IFN-β, IFN-λ, RANTES and other interferon-related factors

## 4 Research status of TIR domain protein in tumor

### 4.1 TIR domain protein and CSCs

CSCs are a small group of cancer cells with abundant stemness and tumor initiation capabilities in tumor microenvironment. CSCs have self-renewal and differentiation capabilities, which can promote tumor progression and metastasis, and lead to treatment resistance and cancer recurrence (89), because of their above properties, they are considered to be the origin cells of tumorigenesis and key drivers of malignancy, so TLR-targeted tumors are inseparable from CSCs.

In studies on TLR-mediated enhancement of CSC stemness, in breast cancer, TLR3 activation drives the conversion of non-CSCs into CSCs by synergistically activating the β-catenin and NF-κB pathways, leading to tumor recurrence. Research teams have found that cardamonin can block this TLR3-induced transformation by simultaneously inhibiting both pathways, thereby eliminating the TLR3-enhanced CSC phenotype (90). Also in breast cancer, TLR2 is overexpressed in CSCs and promotes their self-renewal. Clinical studies indicate a significant correlation between high TLR2 expression and poor prognosis in breast cancer patients (91). TLR4 activation can also induce TWIST1 and facilitate the formation of tumor-initiating stem-like cells in the mouse liver through cooperation with Nanog and STAT3 (92). In lung cancer stem cells, enhanced mitophagy leads to the accumulation of mtDNA (mitochondrial DNA), which upregulates the Notch1-AMPK pathway via TLR9-MyD88 signaling, promoting CSC proliferation and chemotherapy resistance. This suggests that targeting TLR9 can significantly inhibit the tumorigenic capacity of patient-derived organoids (93). In human hepatocellular carcinoma (HCC), increased stem-like traits are associated with TLR4 expression, and the expression of TLR4 in HCC cells closely correlates with their invasive and migratory abilities. In clinical HCC tissues, high TLR4 expression is significantly associated with early recurrence and reduced survival, contributing to poor prognosis in HCC (94). These studies demonstrate that TLR activation enhances cancer cell stemness, providing new therapeutic avenues for targeting cancer stem cells.

Furthermore, CSCs can also mediate their own survival by expressing low levels of TLR signaling molecules or modulating signaling pathways. In hematopoietic stem and progenitor cells (HSPCs) of myelodysplastic syndromes (MDS), TLR-TRAF6 signaling drives a switch in NF-κB signaling from the canonical to the non-canonical pathway by activating the A20 protein. This shift protects MDS HSPCs from inflammatory damage and confers a proliferative advantage, suggesting that inhibiting the non-canonical NF-κB pathway can reverse the competitive survival of CSCs (95). In glioblastoma, CSCs exhibit low TLR4 expression, which enhances retinoblastoma-binding protein 5 (RBBP5) function as a core activator of stemness transcription factors, thereby promoting CSC self-renewal and reinforcing CSC properties. This allows them to survive by disregarding inflammatory signals (96). These findings indicate that targeted upregulation of TLR expression in CSCs may inhibit tumor growth.

### 4.2 TIR domain protein and TME

**Figure 4:**
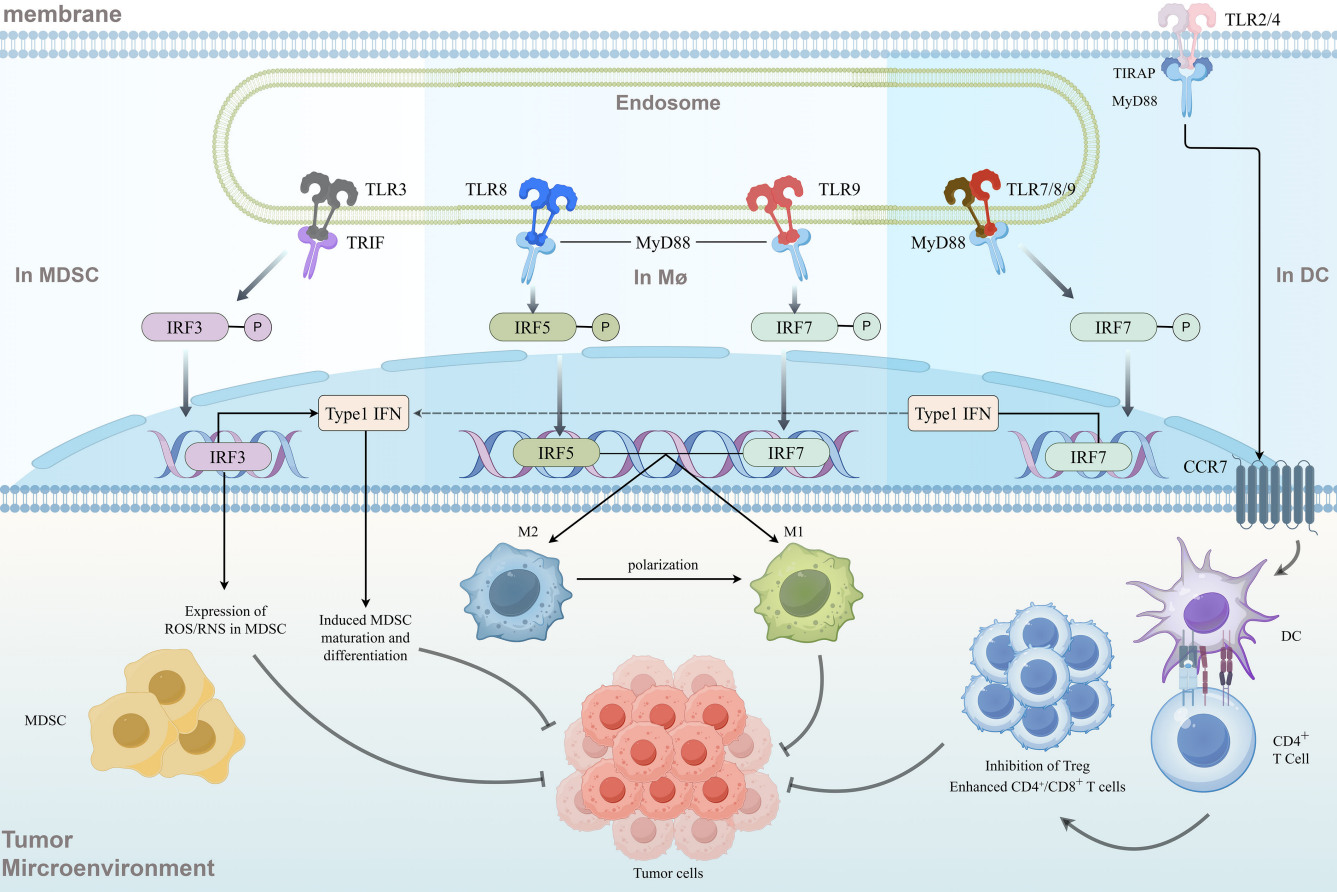
On the cell membrane, TLR2/4 initiate downstream signaling by recruiting the adaptor proteins TIRAP and MyD88, while endosomal membrane-localized TLR3 activates signaling via the adaptor protein TRIF. TLR7/8/9 rely on MyD88 to mediate signal transduction. These TLR subtypes are selectively expressed in immune cells such as MDSCs, macrophages, and DCs. Upon activation, they drive the phosphorylation and activation of interferon regulatory factors (IRF3, IRF5, IRF7), ultimately inducing the secretion of Type I interferons, thereby providing a critical signaling foundation for immune remodeling in the TME. Concurrently, TLR signaling activation in MDSCs can induce the generation of reactive oxygen/nitrogen species (ROS/RNS) and downregulate their immunosuppressive functions. In macrophages, TLR signaling promotes a shift toward an anti-inflammatory phenotype through an IRF5-mediated pathway. In DCs, TLR activation facilitates IRF7-driven Type I IFN secretion and upregulation of the chemokine receptor CCR7, synergistically promoting DC maturation and migration to tumor-draining lymph nodes, thereby enhancing antigen presentation capacity. Furthermore, activation of TLR2/4 and related signaling pathways can reverse the M2 (pro-tumor) phenotype of TAMs, inducing their repolarization toward the M1 (anti-tumor) phenotype and enhancing the secretion of anti-tumor cytokines such as TNF-α and IL-12. This process attenuates the immunosuppressive milieu of the TME while directly augmenting the proliferative activity and cytotoxic function of CD4^+^ helper T cells and CD8^+^ cytotoxic T cells. By downregulating the immunosuppressive effects of Tregs, TLR signaling alleviates their functional suppression of effector T cells. Ultimately, these mechanisms reshape the immunosuppressive TME of an “immunologically cold tumor” into the pro-inflammatory phenotype of an “immunologically hot tumor”. Through the coordinated action of innate and adaptive immunity, tumor cell killing is efficiently enhanced, leading to a significant amplification of anti-tumor immune effects.

The TME, as the extrinsic environment for tumor growth, plays a critical role in cancer progression (97). In the context of human efforts to combat tumors, although tumor vaccines have been developed through clinical research, their efficacy remains very limited, which is partly associated with the defensive role of the TME (98). Recent studies on TLR-TIL have indicated that TLRs, by recognizing DAMPs or PAMPs, play an important role in the TME (99). On one hand, their abnormal activation can promote tumorigenesis through pro-inflammatory signaling; on the other hand, targeting TLRs can remodel the immunosuppressive TME and enhance anti-tumor immunity (34, 98). Targeting TLRs remodeling the immunosuppressive tumor microenvironment to enhance anti-tumor immunity as shown in the (**Figure 4**).

Regarding the molecular mechanisms mediating the reprogramming of innate immune cells, TLR agonists recruit adaptor proteins such as MyD88/TRIF via the TIR domain, activating downstream NF-κB and MAPK pathways and directly regulating macrophage polarization. Studies have shown that mannosylated nanoparticles delivering the TLR7/8 agonist R848 (Man-pD-PLGA-NP@R848) target tumor-associated macrophages (TAMs) through mannose receptor-mediated endocytosis, inducing their transition from the M2 (pro-tumor) to the M1 (anti-tumor) phenotype. Simultaneously, dendritic cells in the TME are activated, enhancing antigen presentation capacity, thereby converting “cold tumors” into “hot tumors” (100). Meanwhile, in an osteosarcoma model, TLR9 agonists remodel the immunosuppressive TME by reducing M2-like macrophages and increasing the infiltration of activated CD8^+^ T cells (101), indicating that TLR signaling can alter cytokine profiles in the TME, thereby influencing APCs, particularly macrophages, and modifying the course of tumor immunity. Furthermore, in immunologically cold breast cancers (e.g., TNBC), mitochondrial complex I inhibitors (e.g., IACS-010759) synergize with TLR agonists (e.g., CpG-ODN) via the TIR-MyD88 pathway to upregulate NADPH oxidase activity in neutrophils, generating large amounts of ROS and enabling them to acquire tumor-killing capabilities independent of CD8^+^ T cells (102). This demonstrates that, in addition to modulating cytokines to act on APCs in the TME, TLR signaling also effectively synergizes with neutrophils.

Furthermore, emerging advances have been made in the synergistic strategies of adaptive immune activation and epigenetic regulation concerning the interplay between TLR and TME. Studies in colorectal cancer have demonstrated that transfection with miR-29b mimics can target the 3’UTR of TLR1/7/8, inhibit their expression, reduce IL-6 secretion, and reverse Treg-mediated immunosuppression, indicating its tumor-suppressive role (103). In contrast, lncRNA H19 acts as a “sponge” for miR-29b, alleviating its inhibitory effect on TLR2 mRNA, upregulating TLR2 expression, and promoting gastric cancer metastasis, suggesting a tumor-promoting function (104). Both cases illustrate that non-coding RNAs (ncRNAs) can precisely target TLR signaling pathways and influence T-cell responses. Additionally, beyond the TLR–ncRNA network-mediated regulation of T-cell responses, we have observed that employing a nano-delivery system co-loaded with TLR agonists to target the tumor microenvironment can enhance the synergistic effects of radiotherapy. Research has shown that polymeric micellar nanoparticles (PMNPs) co-delivering the TLR7/8 agonist (R848) and a PI3Kδ inhibitor (Idelalisib) significantly reduce the accumulation of MDSCs and infiltration of Tregs in a triple-negative breast cancer (TNBC) model, while enhancing the activity of CD4^+^/CD8^+^ T cells and improving the antitumor efficacy of radiotherapy (105). This strategy offers new prospects for addressing the lack of targeted approaches against immunosuppressive MDSCs and Tregs in the TME.

### 4.3 TIR domain protein and immune cell

Significant multidimensional progress has been made in TIR domain-mediated immunoregulation of macrophages. In terms of signaling pathway research, MyD88-dependent TLR signaling (e.g., TLR4/9) drives macrophage polarization toward the M2 phenotype (75). In contrast, TRIF-dependent TLR signaling (e.g., TLR3) promotes M1 polarization and enhances the secretion of iNOS and TNF-α (106). In the field of dietary interventions in cancer therapy, recent studies have found that a high-fat diet activates the TLR4-TIR signaling in adipose tissue macrophages (ATMs), inducing CXCL10 secretion and recruiting CD8^+^ T cells, which significantly suppresses peritoneal metastasis of colorectal cancer (a 68% reduction in metastatic foci). This effect synergizes with chemotherapy in immunocompetent models (107). In tumor immunotherapy, the second-generation CD3ζ-TIR dual-signaling chimeric receptor developed by Lei Anhua’s team significantly enhances antigen-dependent M1 polarization and M2 resistance in CAR-macrophages. By activating the NF-κB pathway, it overcomes the immunosuppressive tumor microenvironment, resulting in a 3.7-fold increase in tumor phagocytosis efficiency compared to conventional CAR-M therapies (108).

Furthermore, targeting TIR to modulate myeloid cells has emerged as a novel strategy in tumor immunotherapy. Beyond its role in TLR pathways, the TIR domain possesses NAD+ hydrolase activity (109), generating cyclic ADPR isomers (such as 2’cADPR and 3’cADPR) that act as second messengers to enhance the antigen-presenting capacity of DCs (17). Concurrently, the combination of IFN-γ with TLR agonists (e.g., TLR2, TLR4, or TLR9 agonists) enhances DC activation and function, thereby augmenting antigen-specific T cell responses (110). Additionally, studies have shown that kinetic stimulation of TLR4, combined with TLR7/8 activation in DCs, induces strong synergistic effects through two consecutive waves of stimulation from the cell membrane to endosomes within an appropriate temporal window, enabling spatiotemporally programmed activation of dendritic cells (111). Research also indicates that the TLR5 ligand flagellin significantly upregulates the expression of maturation markers on CD103^+^ (cDC1) and CD11b^+^ (cDC2) subsets, demonstrating potent efficacy in activating neonatal lung APCs (112). Moreover, Wang Ruonan’s team designed a DC-targeting nanoregulator (DNR) that reprograms dendritic cells in tumor-draining lymph nodes (TDLNs) by synergistically activating TLR4 (via mannan) and TLR7/8 (via an IMDQ prodrug) signaling pathways. This combined tumor vaccine achieved an 80% tumor regression rate in mice and established long-term immune memory (111).

In the context of TIR domain-mediated T-cell immunoregulation in tumors, studies have revealed that TIR domains can modulate T cells through cytokine secretion. Specifically, TLR2-TIR promotes the secretion of IL-6 and IL-23 in CD4+ T cells via the MyD88-dependent pathway, thereby enhancing Th17 differentiation while simultaneously suppressing Treg function (113). Regarding the regulation of CD8+ T cells, agonists of the TLR2-TIR signaling pathway can enhance cell proliferation and IFN-γ production, and influence memory cell formation in CD8+ T cells by reducing costimulatory signals from APCs (114–116). In terms of modulating Treg cells for tumor immunotherapy via the TIR domain, TLR8 has been shown to reverse the immunosuppressive function of Tregs and enhance their anti-tumor activity by inhibiting glycolysis and glucose uptake (117, 118). In the B16-OVA melanoma model, local administration of TLR7/8 agonists polarizes Th1 responses, affecting anti-tumor immunity, and activates NK cells and CD8+ T cells (119). In tumor models of lung cancer, leukemia, and melanoma, TLR1/2 antagonists suppress the inhibitory function of Foxp3+ Tregs, enhance the cytotoxicity of tumor-specific CTLs, and lead to the depletion of tumor-infiltrating Treg cells (120, 121). Additionally, recent studies in mouse models have demonstrated that TLR5 agonists inhibit liver metastasis and promote the formation of CD8+ T memory cells (122). These findings collectively highlight the broad potential of TLR agonists or antagonists in T cell-based cancer therapy.

Furthermore, we observed novel insights regarding TLRs in NK cell research. Studies on TLR expression in NK cells indicate that NK cells pecifically express TLR1, 2, 3, and 7 (123). In gastric cancer (GC) patient models, compared to healthy volunteers, the expression of TLR-2, TLR-3, TLR-4, and TLR-9 was significantly elevated in GC patients, with higher TLR expression observed in more advanced GC subtypes (124). This suggests that cancer can alter TLR expression in NK cells, and different cancer subtypes may exhibit distinct TLR expression profiles, indicating the potential of TLRs as biomarkers for cancer diagnosis, subtyping, and prognosis evaluation. In terms of TLR ligand-targeted modulation of NK cells, TLR8 agonists (e.g., R848) and TLR9 agonists (e.g., ODN2006) can respectively regulate immunomodulatory and cytotoxic NK cells, increase the expression of activation receptors such as NKG2D and NKp44, and synergistically enhance anti-tumor activity (125). Similarly, CAR133-NK92 cells equipped with TLR5 agonists have been shown to specifically eliminate CD133-positive colon cancer cells in a CAR133-dependent manner, demonstrating that TLR agonist-mediated regulation of NK cells can exert anti-tumor effects (126). These findings, together with the aforementioned regulatory roles of TLR agonists on other immune cells, reveal the significance of TLR agonists in targeting immune cells for cancer therapy.

Interestingly, we noted that in *C. elegans*, TIR-1 is specifically expressed on the surface of lysosome-related organelles (LROs) in intestinal cells, enabling these cells to monitor host damage caused by bacterial effector proteins. This may provide new insights for future TIR-1-targeted strategies in intestinal tumors (127).

### 4.4 TIR domain protein as NADase enzymes

TIR domain proteins have long been considered protein-protein interaction scaffolds in innate immune signal transduction. However, recent studies have revealed that their functions extend far beyond this role. The TIR domain is highly evolutionarily conserved, widely distributed in both prokaryotes and eukaryotes, and exhibits enzymatic activity capable of degrading NAD^+^, referred to as NADase activity (8, 128). This discovery has expanded our understanding of the functional diversity of TIR domains and uncovered their non-canonical mechanisms in immune signal transduction.

Research on the enzymatic activity of the TIR domain is particularly advanced in the plant immune system. Studies indicate that the TIR domains of plant immune receptors e.g., RPP1-TIR can function as NAD^+^ hydrolases, catalyzing the production of 2’,3’-cyclic adenosine monophosphate (2’,3’-cAMP) and 2’,3’-cyclic guanosine monophosphate (2’,3’-cGMP). These cyclic nucleotides act as immune signaling molecules that directly mediate cell death, representing a key mechanism in plant disease resistance immunity (12, 129–131). This finding not only reveals a novel synthetase function for TIR domains in plants but also suggests a potentially conserved enzymatic activity mechanism in eukaryotes. In microbial systems, TIR domains similarly demonstrate NADase activity. For instance, bacterial TIR domain proteins such as ThsB can catalyze NAD^+^ cleavage, generating cyclic ADPR derivatives e.g., 2’cADPR and 3’cADPR that participate in host defense responses as immune signaling molecules (132, 133). These cyclic ADPR molecules serve as second messengers in microbial immunity, activating downstream immune pathways and illustrating the functional conservation of TIR domains across species in immune systems (17).

In contrast, research in mammalian systems remains a knowledge gap. While the enzymatic functions of TIR domains and the immune messenger molecules they generate in mammalian systems remain a frontier awaiting exploration, this area offers a novel perspective for understanding the tumor immune microenvironment. Within the TME, persistent inflammation and cell death may lead to NAD^+^ level fluctuations and the accumulation of potential immune messengers. An innovative hypothesis can thus be proposed: TIR domain proteins in tumor cells or tumor-infiltrating immune cells (such as MDSCs or Tregs) may utilize their NADase activity to deplete local NAD^+^ pools or generate immunomodulatory metabolites, thereby shaping an immunosuppressive microenvironment. For instance, certain cyclic ADPR derivatives could potentially regulate metabolic reprogramming or functional states of immune cells. Consequently, targeting the enzymatic activity of TIR domains—whether by developing agonists to enhance anti-tumor immunity or inhibitors to block their tumor-promoting functions—represents a highly promising new class of immunotherapy strategies. Future research should prioritize the identification of specific immune messengers produced by mammalian TIR proteins and elucidate their precise roles in tumor initiation and progression.

## 5 The potential of TIR domain proteins in cancer immunotherapy

### 5.1 Therapy strategy and key drug of TIR pathway agonist

The TIR signaling pathway, primarily through its two major branches—the MyD88-dependent and TRIF-dependent pathways—regulates inflammatory responses, immune cell infiltration, and tumor cell biological behavior. The application of TLR in tumor immunotherapy is summarized in (**Table 4**). Its application in cancer therapy mainly involves agonists and related formulations; below, we first introduce the agonist component.

**Table 4:** Application of TLRs in cancer immunotherapy.

Class	Targets	Representative agents	Core mechanism	Experimental results/application	Clinical status	References
Agonist	TLR2	CADI-05	Targets DSC3-positive cancer cells, activates the TLR2, and remodels the antitumor immune microenvironment	advanced NSCLC	With cisplatin-paclitaxel: Phase 1b/2 trial	(138)
	TLR3	Poly I:C	Activates TRIF pathway, induces IFN-1, and enhances DC cross-presentation	used in combination with cancer vaccines as an immune adjuvant for advanced or recurrent esophageal cancer	Phase I trial	(244, 245)
	TLR4	MPLA	Drives DC maturation, enhances CD8^+^T infiltration	sLNPs-OVA/MPLA: EG.7-OVA tumors	Preclinical trial	(246)
	TLR7/8	IMIQ R848	Activates intratumoral DCs and TILs; Promotes IFN-α/TNF-α secretion	IMIQ: basal cell carcinoma, oral squamous cell carcinoma R848: pancreatic ductal adenocarcinoma (PDAC)	IMIQ: approved R848: Phase I/II	(143, 144, 227, 247)
	TLR9	CpG ODN	Activates MyD88, enhances Th1/CTL response	With Trastuzumab:metastatic HER2+Breast Cancer melanoma	With Trastuzumab: Phase I melanoma: Phase I/II	(154, 156, 197, 248, 249)
Inhibitor	TLR4	TAK-242	Blocks TLR4, inhibits NF-κB	+fulvestrant: NSCLC inflammatory disease research	Preclinical	(168, 169)
	MyD88	ST2825,TJ-M2010-2	Blocks MyD88, inhibits NF-κB, reduces M2 macrophages	ST2825:inflammation TJ-M2010-2:breast cancer	ST2825: preclinical TJ-M2010-2: preclinical	(165, 170, 175)
	TBK1	Amlexanox	Inhibits AKT/NF-κB, induces G1 arrest, downregulates EMT	endometrial cancer	preclinical	(176)

The core of agonist therapeutic strategies lies in activating the immunostimulatory branches of the TIR signaling pathway to enhance anti-tumor immune responses. The MyD88 and TRIF pathways, as the two core branches of TIR signaling (29, 134–136), play complementary roles in immune activation, providing clear targets for agonist design. Activation of the NF-κB and MAPK signaling axes via the MyD88 pathway (137) promotes dendritic cell (DC) maturation and antigen presentation function, laying the foundation for enhancing anti-tumor immune responses. The following section details clinical research and applications of TLR agonists.

TLR2 activates the downstream MyD88-NF-κB signaling pathway by recognizing exogenous PAMPs and DAMPs. In a randomized controlled trial conducted by Belani et al., the efficacy and safety of the TLR2 agonist CADI-05 combined with cisplatin-paclitaxel as first-line therapy for advanced non-small cell lung cancer (NSCLC) were evaluated. The study demonstrated that while the addition of CADI-05 did not significantly improve overall survival in the intent-to-treat population, the squamous cell carcinoma subgroup showed a significant prolongation of median overall survival by 127 days (HR 0.55, P = 0.046). The combination therapy was well-tolerated without increased systemic adverse reactions. These findings indicate the potential immunotherapeutic value of TLR2 agonists in desmocollin-3-expressing squamous NSCLC (138).

The TLR4 agonist Monophosphoryl Lipid A (MPLA), a derivative of Lipopolysaccharide (LPS), significantly upregulates the expression of co-stimulatory molecules such as CD80 and CD86 on DCs, enhancing the cross-presentation efficiency of Tumor-Associated Antigens (TAAs), thereby increasing T cell infiltration (139). According to research by Sun L et al., in a 4T1 breast cancer mouse model, the number of CD8+ cytotoxic T cells in the tumor tissue of the MPLA + IFNγ combination treatment group increased significantly, with the infiltration density approximately more than 2-fold higher than the control group (untreated). In contrast, the IFNγ monotherapy group showed a smaller increase in CD8+ T cell infiltration (approximately 1.2-fold higher than control) and a lower degree of T cell activation compared to the combination group. Around day 21 post-tumor inoculation, the tumor volume in the combination treatment group was reduced by approximately 60% compared to the control group, whereas the IFNγ monotherapy group showed only about a 30% reduction, clearly demonstrating the synergistic effect of the combination therapy (140).

TLR7 primarily recognizes viral single-stranded RNA (141), and its agonists are being actively investigated in clinical trials for patients with advanced or metastatic cancer (142). TLR7 agonists stimulate immune cells to secrete various cytokines, such as IFN-α and TNF-α, thereby enhancing the body’s anti-tumor capacity (143). In existing research, a small molecule TLR7 agonist was conjugated to the MUC1 glycopeptide and the carrier protein BSA to form a three-component conjugate (BSA-MUC1-TLR7A), which was combined with alum adjuvant. The results indicated that the alum adjuvant and the built-in TLR7 agonist synergistically enhanced the anti-MUC1 antibody response, induced a Th1-biased immune response, and also boosted the MUC1 glycopeptide-specific memory CD8+ T cell immune response, significantly inhibiting tumor growth and prolonging mouse survival (144). In a recent clinical trial (NCT04883645), the local TLR7 agonist imiquimod was evaluated as a neoadjuvant therapy in patients with early-stage oral squamous cell carcinoma (OSCC). Among the 15 patients treated with 5% cream once daily for 28 days prior to surgery, 60% (9/15) achieved an immune-related major pathological response, defined as residual viable tumor cells of less than 10%, including two cases of pathological complete response. With a median follow-up of 17 months, the 1-year recurrence-free survival rate reached 93%, and the treatment was well tolerated. These results support the use of local TLR7 agonists as an effective neoadjuvant immunotherapy strategy for early-stage OSCC (145).

Additionally, clinical studies in glioblastoma have demonstrated that the application of TLR7/8 agonists combined with GSM DHCR7/cholesterol signaling modulation significantly promotes the polarization of glioblastoma stem-like cells (GSMs) toward an anti-tumor phenotype and improves the TME. This combination strategy exhibited superior anti-tumor efficacy in both orthotopic glioblastoma models and postoperative recurrence models (146). Furthermore, researchers have developed a β-cyclodextrin nanoparticle (CDNP) formulation encapsulating the TLR7/8 agonist R848 (CDNP-R848), which reprograms myeloid cells in the glioma microenvironment to induce regression of syngeneic experimental gliomas and significantly improve survival rates (147). In the field of hepatocellular carcinoma (HCC) therapy, clinical research on TLR7/8 agonists also warrants attention. Studies have shown that the TLR7 agonist Imiquimod inhibits the proliferation and mammosphere formation of hepatic cells and stem cells, while reducing the stem cell population. These effects are potentially mediated through the TLR7/IκB kinase/nuclear factor-κB/interleukin-6 signaling pathway, accompanied by downregulation of Snail expression levels (148). Moreover, combination therapy employing NK cells pre-activated with dendritic cells and the TLR7/8 agonist gardiquimod (GDQ) significantly suppresses the growth of human HepG2 liver carcinoma xenografts (149). Notably, in clinical investigations of head and neck squamous cell carcinoma (HNSCC), multiple studies have evaluated TLR8/9 agonists—including the TLR8 agonist motolimod and TLR9 agonists SD-101, IMO-2055, and EMD1201081—in combination with cetuximab or PD-1 inhibitors. Analysis revealed that in Phase II clinical trials, the TLR agonist arms showed no significant improvements in objective response rate or median progression-free survival compared to control groups. Furthermore, some agents led to early trial termination due to serious toxicity. Overall, current evidence does not support a substantial clinical benefit of TLR agonists in recurrent or metastatic HNSCC, indicating limited therapeutic potential. Future efforts should focus on optimizing combination strategies to enhance efficacy (150).

TLR9 recognizes unmethylated CpG DNA sequences of bacterial or viral origin (151–153). In cancer immunotherapy, CpG Oligodeoxynucleotides (CpG ODN) are widely studied as TLR9 agonists. In existing research for melanoma, a tumor vaccine composed of Class C CpG ODN and irradiated tumor cells triggered significant long-term anti-tumor immune responses *in vivo*. CpG ODN binds to TLR9 on the surface of Antigen-Presenting Cells (APCs), triggering downstream signaling pathways: upregulation of MyD88 gene expression, subsequently initiating the differentiation and maturation of APCs (154). Earlier research showed that vaccination with apoptotic tumor cells covalently conjugated with immunostimulatory CpG ODN triggered the expansion of tumor-specific Cytotoxic T Lymphocytes (CTLs), thereby reducing the growth of established tumors and preventing metastatic spread (155). In lung cancer research, a co-delivery system for CpG ODN and a PD-L1 antagonistic peptide based on mannose-modified liposomes (HA/M-Lipo CpG-P) was constructed. Results demonstrated that the HA/M-Lipo CpG-P complex successfully reprogrammed M2-type macrophages to M1-type within the tumor microenvironment, thereby activating anti-tumor immune cells and inhibiting tumor growth, showing better anti-tumor efficacy than monotherapies (156). Beyond this application, the immune exosomes loaded with self-assembled nanomicelles and functionalized with anchored TLR9 agonist CpG oligonucleotides (CpG-EXO/TGM) have also been employed for the delivery of temozolomide to achieve enhanced anti-GBM efficacy (157). Moreover, a targeted lipid nanovaccine, constructed by co-delivery of tumor-specific neoantigens via a cholesterol-conjugated TLR9 agonist cationic liposome preparation LNPsD18, significantly inhibits tumor growth by remodeling TME, thereby improving *in situ* Survival of HCC and colorectal cancer models (158).

TRIF pathway agonists, on the other hand, initiate adaptive immune responses by inducing a Type I interferon response (159). Among them, the TLR3 agonist Polyinosinic-polycytidylic acid (Poly I:C), a classic TRIF pathway activator, can directly stimulate tumor cells to release Immunogenic Cell Death (ICD)-associated molecules like HMGB1 (160), while also promoting IFN-I production by DCs, enhancing cross-priming effects. In lung adenocarcinoma (LUAD) treatment experiments, Poly I:C upregulated PD-L1 expression by activating the NF-κB signaling pathway, while simultaneously enhancing immune cell infiltration, antigen presentation, and immune activation within the tumor immune microenvironment. Ultimately, this increased the sensitivity of LUAD to PD-1/PD-L1 inhibitors and suppressed tumor proliferation, migration, and invasion (161).

### 5.2 Therapeutic strategies and key drugs of TIR pathway inhibitor

Next, we present the section on inhibitors. The core strategy of inhibitor therapy focuses on blocking pro-tumorigenic branches of TIR signaling to suppress tumor progression, particularly targeting key molecules and interactions associated with tumor metastasis (162), angiogenesis (163), and immunosuppression (164).

Major targets include members of the TLR family. Among them, TLR4 is a extensively studied pro-tumorigenic target. Inhibitor design against TLR4 often targets its ligand-binding domain or intracellular signaling region to prevent the binding of ligands like Lipopolysaccharide (LPS) or the initiation of downstream signaling (165). Sustained activation of TLR4 signaling promotes tumor metastasis through multiple mechanisms. In breast cancer models, high expression of TLR4 on tumor cells (166) leads to activation of the MyD88/NF-κB pathway upon recognition of HMGB1 in the tumor microenvironment, inducing the secretion of Matrix Metalloproteinase-9 (MMP-9) and Vascular Endothelial Growth Factor (VEGF), thereby enhancing tumor cell invasiveness and angiogenic potential (167). Preclinical studies have shown that after chronic UVB exposure, compared to the control group, mice treated with the TLR4 inhibitor TAK-242 exhibited a significant reduction in CD4^+^CD25^+^ regulatory T cells in the spleen and lymph nodes, decreased expression of Foxp3 and IL-10 in tumor tissue, markedly inhibited skin carcinogenesis, and significant downregulation of pro-inflammatory cytokines IL-1β, IL-6, and TNF-α (168). In primary non-small cell lung cancer (NSCLC) tissues, both fulvestrant and TAK-242 monotherapies inhibited the migration and invasion of NSCLC cells, with the combination showing the strongest inhibitory effect. Furthermore, TAK-242 helped fulvestrant restrict the formation and function of invasive pseudopodia in NSCLC cells (169).

Beyond cell surface receptors, intracellular adaptor proteins MyD88 and TRIF are also critical targets. Inhibiting the interaction between MyD88 and TLRs or its homodimerization can block downstream inflammatory pathways, as seen with the MyD88 inhibitor ST2825 (170). Targeting the binding sites of TRIF with TRAF6 or TBK1 can suppress IFN-related pro-tumorigenic signaling (171). Additionally, downstream kinases such as IRAK1/4, TAK1, TBK1, and transcription factors like NF-κB are also targeted using small molecule inhibitors to block their activity and disrupt the signal cascade (172–174). The design of these targets aims to precisely inhibit the pro-tumorigenic effects of the TIR pathway while minimizing disruption to host immune defense, providing specific strategies for cancer treatment. Relevant research found that the MyD88 inhibitor TJ-M2010–2 significantly inhibited the proliferation, migration, and invasion of breast cancer cells *in vitro*. Its core mechanism involves inhibiting the dimerization of the TIR domains of two MyD88 molecules, thereby blocking the activation of downstream MyD88/GSK-3β and MyD88/NF-κB signaling pathways, which subsequently affects the activity of related signaling molecules and the expression of cytokines and genes. In *in vivo* mouse models, TJ-M2010–2 not only significantly suppressed tumor growth but also reduced cytokine secretion within the tumor microenvironment and decreased the infiltration of M2-type macrophages, thereby improving the tumor microenvironment to inhibit tumor progression (175).

Mechanistically, the TBK1 inhibitor amlexanox suppresses endometrial cancer cell proliferation, arrests the cell cycle, reduces the expression of epithelial-mesenchymal transition (EMT)-related proteins, and inhibits cell migration by modulating the AKT/NF-κB signaling pathway. *In vivo* experiments using nude mice, both amlexanox treatment and TBK1 knockdown significantly inhibited xenograft tumor growth, and also reduced the levels of EMT-related proteins as well as phosphorylated AKT and NF-κB in the tumor tissues (176).

### 5.3 Synergistic strategies combining TLR agonists with ICI

**Figure 5:**
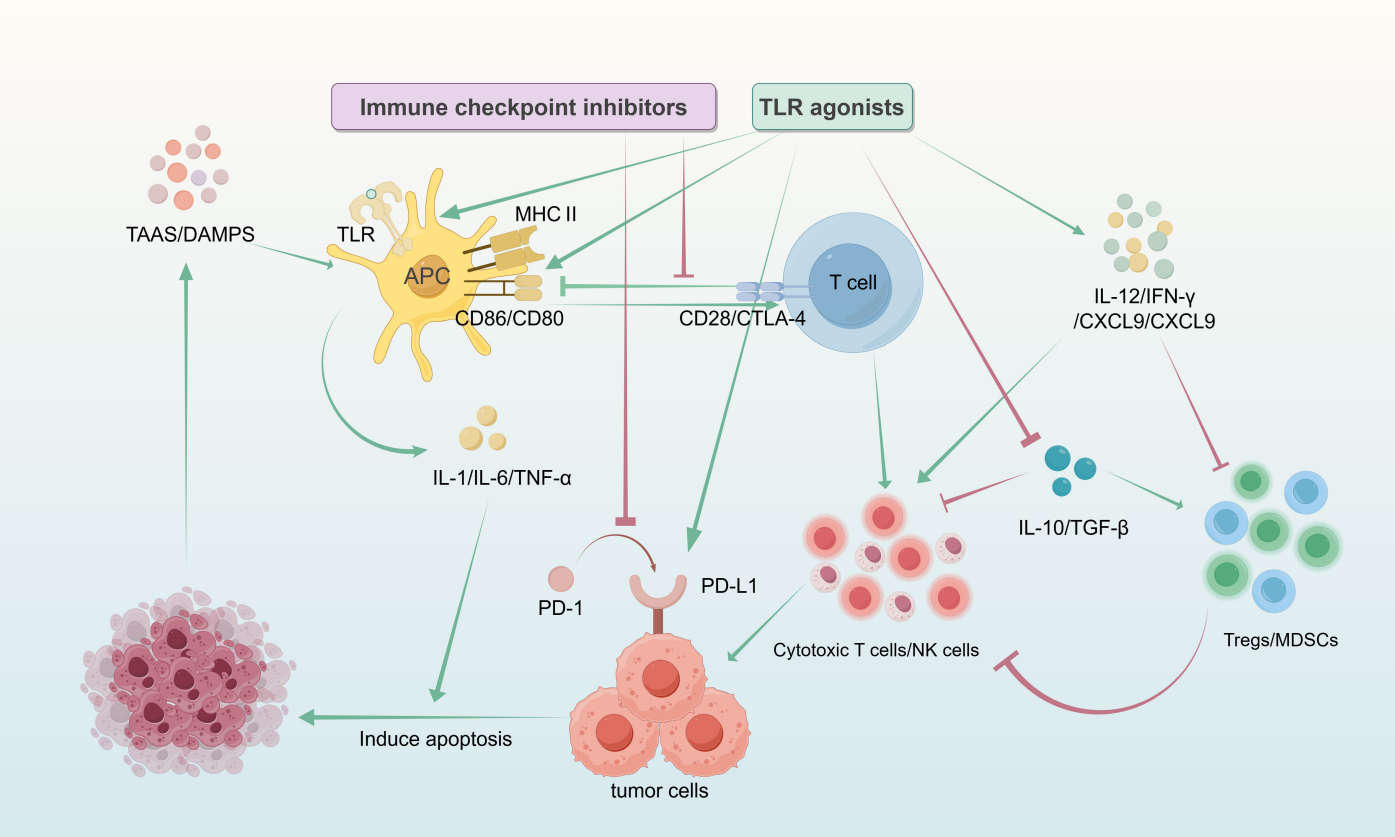
TLR agonists are taken up by APCs, primarily DCs, within the tumor microenvironment. DCs recognize the agonists via endosomal membrane TLRs (such as TLR3, TLR7/8, TLR9) or cell surface TLR4, leading to their activation and maturation. This is characterized by a significant increase in the surface expression of MHC-antigen peptide complexes and co-stimulatory molecules (CD80/CD86). The mature DCs then migrate to the tumor-draining lymph nodes, where they activate naïve T cells through the engagement of the MHC-antigen peptide complex with the T-cell receptor (TCR), combined with the binding of CD80/CD86 to CD28 on the T cell surface. This interaction provides the dual signals necessary for T cell activation, triggering massive clonal proliferation and differentiation of naïve T cells into tumor-specific effector T cells. The activated effector T cells circulate to the tumor tissue and, upon recognizing MHC-antigen peptide complexes on the surface of tumor cells via their TCR, initiate an attack. Simultaneously, the mechanism of immune checkpoint inhibitors is to release T cell inhibition. Tumor cells often upregulate PD-L1, which binds to PD-1 on T cells, transmitting an inhibitory signal that leads to T cell exhaustion. Anti-PD-1/PD-L1 antibodies (ICIs) block this interaction, thereby releasing the “brakes” on T cells and restoring their cytotoxic function. Furthermore, TLR agonists can also induce immunogenic cell death (ICD) in tumor cells, leading to the release of more TAAs and DAMPs. This process further enhances the antigen presentation efficiency of DCs, expands the clonal repertoire of tumor-specific T cells, and ultimately achieves efficient clearance of tumor cells.

Immune Checkpoint Inhibitors (ICIs) enhance disease control by blocking the interaction between immune checkpoints (e.g., PD-1/CTLA-4) and their ligands on antigen-presenting cells or tumor cells, thereby disrupting inhibitory or stimulatory signals and activating cytotoxic T cells (177, 178). However, the clinical efficacy of ICI monotherapy is often suboptimal due to limited response rates (objective response rates), variable efficacy across cancer types, and the development of resistance in some patients (179). The addition of adjuvants to ICI regimens can modulate the intensity of immune responses (180). TLR agonists represent one such class of adjuvants. Their ability to upregulate PD-L1 (181), increase the intratumoral infiltration of cytotoxic T cells, NK cells, and antigen-specific interferon (IFN)-secreting effector cells (182–184) makes them a potential strategy for enhancing the efficacy of PD-1/CTLA-4 inhibitors (119). Exploration of the combination of TLR agonists and ICIs is intensifying, driven by their significant synergistic potential, making the investigation of combination regimens crucial for optimizing cancer immunotherapy. Its process and mechanism are shown in the (**Figure 5**). The following sections detail the mechanisms underlying the enhanced anti-tumor effects observed when these two modalities are combined.

First, TLR agonists can enhance the interaction between APCs and T cells when combined with ICIs. While CTLA-4 blockade alone enhances T cell activation, the concurrent use of a TLR agonist can further amplify this effect by increasing the expression of co-stimulatory molecules (e.g., CD86) and MHC class II on APCs (185). Additionally, TLR activation on DCs and other APCs induces the production of numerous inflammatory cytokines, such as IL-1, IL-6, and TNFα (186, 187). Thus, the combination of TLR agonists and CTLA-4 blockade acts through both direct and indirect mechanisms.

Furthermore, synergy arises from their respective effects on the tumor microenvironment (TME). The TME is often immunosuppressive, characterized by the presence of Tregs, M2-type macrophages, and MDSCs, which hinder T cell infiltration and function (188–190). Combining TLR agonists with ICIs can synergistically counteract this immunosuppression. TLR agonists activate innate immune signaling pathways (e.g., NF-κB, IRF pathways) (191), inducing the secretion of pro-inflammatory cytokines (e.g., IL-12, IFN-γ) and chemokines (e.g., CXCL9, CXCL10) (192, 193). This promotes the migration of effector T cells (CD8^+^ T cells) into tumor tissue and reduces the infiltration of Tregs and MDSCs (105). ICIs then function to release the “brakes” on these T cells within the TME (194, 195). Concurrently, the inflammatory environment induced by TLR agonists can enhance T cell sensitivity to checkpoint blockade, thereby augmenting the efficacy of ICIs (58, 59).

Regarding clinical evidence of synergy, studies in mouse cancer models have demonstrated that the compound D18, when combined with different ICIs, significantly promotes T cell activation and expansion by activating the TLR7/8 signaling pathway, ultimately increasing the overall infiltration of activated CD8^+^ T cells in tumor tissue (196, 197). In Hepatocellular Carcinoma (HCC) research, TLR9 agonists have been shown to upregulate PD-L1 expression, which can ultimately induce immune escape. However, combination therapy with anti-PD-1 or anti-PD-L1 antibodies in HCC models demonstrated far superior anti-tumor efficacy and greater reduction of immunosuppression compared to either agent alone (198). It is noteworthy that an imbalance of cytokines in the TME is one reason for resistance to PD-1/PD-L1 inhibitors (199). TLR agonists can modulate cytokine secretion and remodel the immune microenvironment (200–202). For instance, the TLR9 agonist CpG-ODN promotes dendritic cell secretion of the pro-inflammatory cytokines IL-12 and IFN-γ (192). Combining cytokine-modulating TLR agonists with PD-1/PD-L1 inhibitors can therefore help mitigate resistance to the latter, achieving better therapeutic outcomes.

Simultaneously, regarding their impact on T cell immune responses, TLR agonists can induce immunogenic cell death (ICD) in tumor cells, leading to the release of Tumor-Associated Antigens (TAAs) and Damage-Associated Molecular Patterns (DAMPs). These molecules are recognized by APCs, which cross-present them to T cells, thereby activating a broader repertoire of tumor-specific T cell clones (203, 204). For example, TLR4 agonists can enhance ICD in tumor cells; the released TAAs are presented by DCs, activating naïve T cells (205). ICIs then prevent these newly activated T cells from being suppressed by immune checkpoint molecules (e.g., PD-L1) on tumor cells, thereby expanding the anti-tumor T cell population and enhancing killing efficiency (206). When used together, the two drug classes can also activate cross-reactive immune responses and broaden the anti-tumor T cell repertoire.

In the clinical research context of TLR agonists, immune checkpoint inhibitors, and radiotherapy, Giuseppe C et al. conducted a study comparing the TLR7 agonist LHC165 as a monotherapy with its combination with the PD-1 inhibitor spartalizumab (PDR001) in patients with advanced solid tumors. The results showed that the combination regimen demonstrated an acceptable safety profile and preliminary evidence of anti-tumor activity, while also reporting one case of Grade 3 treatment-emergent adverse event (TRAE) of pancreatitis associated with the combination therapy (207). Another study investigated a Phase I clinical trial of the TLR7/8 dual agonist immunomodulator BDB001 combined with the PD-1 monoclonal antibody atezolizumab for the treatment of advanced solid tumors. The results indicated that intravenous administration of BDB001 in combination with pembrolizumab was well-tolerated and, supported by strong systemic immune activation, demonstrated significant clinical efficacy. However, this combination therapy also led to three Grade 3 adverse events, including fatigue, rash, stomatitis, and elevated alkaline phosphatase (208). These findings suggest the potential of combining TLR agonists with immune checkpoint inhibitors for the treatment of solid tumors. However, the adverse events induced by such combinations still require further investigation for resolution.

Meanwhile, preclinical and clinical studies in recent years on the synergistic effects of TLR agonists and radiotherapy have shown promising prospects. Han X et al. applied a cholesterol-conjugated TLR7 agonist liposome in combination with radiotherapy in a mouse model, demonstrating that this combined strategy significantly inhibited tumor growth and induced anti-tumor immune responses (209). Furthermore, a Phase I/II clinical trial in patients with metastatic breast cancer indicated that the combination of local radiotherapy with topical TLR7 agonist application was not only safe and feasible but also induced systemic tumor responses in some patients, with a local objective response rate significantly higher than that of TLR7 agonist monotherapy (210). Additionally, in refractory tumors such as pancreatic cancer, the combination of a TLR7/8 agonist with stereotactic body radiotherapy achieved a disease control rate of 38% in advanced patients, with no treatment-related deaths reported (211). These studies reveal the promising potential of combining TLR agonists with radiotherapy in cancer treatment. However, it is also observed that many combination strategies still lack sufficient clinical trial data or remain confined to animal model studies, indicating that this approach requires further expansion and supplementation with safety assessments in humans.

### 5.4 Enhancing CAR-T immunotherapy through the TIR signaling pathway

In CAR-T cells therapy, integrating TLR-related signaling components (such as TIR domain of TLR2 and MyD88) into the structure of conventional CARs can provide additional costimulatory signals to CAR-T cells, thereby significantly enhancing their functions. In a study (212), enhanced CAR-T cells were constructed by integrating key molecules of the TLR4 signaling pathway(including a truncated TLR4 variant (Δ569TLR4), MyD88, and TRIF) into the structure of a CD19-targeting CAR. *In vitro* experiments demonstrated that these enhanced CAR-T cells exhibited increased cytotoxicity against CD19-positive lymphoma and solid tumor cells, as well as elevated secretion of antitumor cytokines. In NOD scid gamma (NSG) mouse xenograft models, the enhanced CAR-T cells achieved better tumor control (with no tumor recurrence observed for 50 days in the solid tumor model) and showed favorable safety profiles.

Relevant studies have also been conducted on the TIR domain of TLR2 (213). By appending the TIR domain of TLR2 to the 3’ end of the CD3ζ signaling domain in CARs, two novel T cell populations were generated: 1928zT2 T cells, which target CD19, and m28zT2 T cells, which target mesothelin. Green fluorescent protein (GFP)-expressing T cells (GFP T cells) and conventional CAR-T cells (1928z T cells/m28z T cells) were used as controls. *In vitro* experiments showed that both 1928zT2 T cells and m28zT2 T cells (with the TIR domain integrated) exhibited higher killing percentages against target tumor cells, increased secretion of IL-2, IFN-γ and GM-CSF, and better expansion upon serial stimulations compared to control cells, even at low effector to target (E/T) ratios. In *in vivo* studies, 1928zT2 T cells demonstrated more effective elimination of CD19^+^ leukemia cells, reduced tumor burden, and prolonged survival in leukemia models (cell line-derived xenografts and patient-derived xenograft models); meanwhile, m28zT2 T cells could shrink mesothelin^+^ solid tumors and inhibit tumor metastasis in solid tumor models—whereas conventional m28z T cells led to increased tumor burden in metastatic models. In a phase I clinical trial (NCT02822326), one patient with relapsed and refractory B-cell acute lymphoblastic leukemia (B-ALL) received a single dose of 1928zT2 T cells at 5×10^4^ cells/kg, resulting in complete eradication of leukemia cells. Subsequently, three additional patients with relapsed or refractory B-ALL were treated with 1928zT2 T cells, all achieving complete remission without serious adverse events.

In addition, the integration of TLR-related signaling components into chimeric antigen receptors CARs can also ameliorate T cell exhaustion. In the aforementioned experiments (213), conventional m28z CAR-T cells exhibited a significant increase in the expression of the exhaustion marker TIM-3 following repeated stimulations; in contrast, m28zT2 cells (harboring TLR2) showed significantly lower TIM-3 expression levels compared to the m28z group, with no significant differences in the expression of PD-1 or LAG-3. These results indicate that TLR2 signaling can specifically alleviate the exhaustion process of CAR-T cells. Another research team (214) specifically delivered a fluorescein-conjugated TLR7 agonist to CAR-T cells. *In vitro* experiments demonstrated that low concentrations of fluorescein-TLR7-1A restored the function of exhausted CAR-T cells—whose cytotoxic efficiency had decreased to 15% following three rounds of stimulation. Specifically, the cytotoxic efficiency of these exhausted CAR-T cells was restored to over 50%, while the proportion of PD-1^+^TIM3^+^LAG3^+^ cells (a hallmark of T cell exhaustion) was reduced by 40%–50%. Notably, this targeted delivery approach avoided the non-targeted activation issue commonly associated with free TLR7-1A.

Notably, such approaches are not without potential issues: the ability of TLR signaling to enhance cytokine production in T cells may increase the risk of cytokine release syndrome (CRS). If this risk can be avoided, this method will be further refined.

### 5.5 Challenges, research gaps, and emerging issues in clinical translational medicine

Although TLRs hold significant potential in cancer immunotherapy by activating the host immune system to combat tumor cells, their clinical translation still faces considerable challenges.

Targeted delivery efficiency represents the foremost challenge. For TLR agonists to exert their therapeutic effects, they must be precisely delivered to immune cells within the TME. In conventional delivery approaches, such as encapsulation within liposomes, TLR agonists are readily recognized and cleared by the mononuclear phagocyte system (MPS) during systemic circulation, thereby preventing effective accumulation within tumor tissue. For example, when some related experiments studied the delivery of TLR agonists by PEG-ylated liposomes, they found that even after PEG modification (the “stealth” strategy), the production of anti-PEG antibodies will still be induced after repeated administration (215), it was reported that in the human repeated administration model, the average elevation of anti-PEG IgG and IgM reached 13.1-fold and 68.5-fold, respectively, and these antibodies accelerate the production of liposomes by MPS through opsonization as well as activation of the complement system (216), resulting in drug accumulation at tumor sites in human patients as low as approximately 0.5% ID/kg (217). However, in recent years, novel nanocarriers have continuously emerged and demonstrated considerable potential. Nanocarrier systems can effectively prevent the premature degradation of antigens and adjuvants, as well as their unnecessary dispersion before reaching the target site (218). Recent studies have designed poly(propylene sulfide) nanoparticles (PPS NPs) for the delivery of TLR7/8 agonists. By employing different linkers to covalently conjugate the agonists to PPS NPs, the alkyl-linked nanoparticles exhibited the optimal performance, selectively prolonging the activation duration of DCs while reducing overactivation of other immune cells such as macrophages. In the EG.7-OVA tumor mouse model, the study demonstrated significant delay in tumor growth, extended survival, and concurrently induced the infiltration and activation of antigen-specific CD8^+^ T cells within the TME, thereby improving targeted delivery efficiency (219). It is important to note that the Biocompatibility of nanocarriers is a key consideration for clinical translation. Many nanomaterials, such as some inorganic nanoparticles, may have problems such as long-term *in vivo* retention, potential immunogenicity, or unpredictable toxicity of degradation products (220–222). Therefore, the development of materials with good biodegradability and low toxicity, such as PPS NPS, is crucial and requires systematic evaluation of their hemocompatibility, tissue distribution, and clearance kinetics in preclinical studies.

Systemic toxicity remains another critical concern. When TLR agonists are administered systemically, they may excessively activate the immune system and trigger severe adverse effects such as cytokine release syndrome (223). Notably, there are important differences in TLR signaling between mice and humans, such as the stronger agonistic activity of the TLR7/8 agonist R848 against human TLR8 than against Mouse Tlr8 (224, 225); Whereas the function of mouse TLR13 has no direct homolog in humans (226), these differences may lead to incomplete extrapolation to humans of the safety and efficacy data observed in mouse models, as well as a lack of understanding of the role of TLR13 in humans. In this context, a recent study leveraged single-oxygen atom engineering to develop a radiotherapy-responsive prodrug platform technology (designated as SAE-RAP). To address the toxicity issues associated with the TLR7/8 agonist IMDQ, a radiotherapy-responsive small-molecule TLR7/8 agonist prodrug, O-R848, was developed. It is in a “silent” state after entering the body, and the agonist technical drug is released only during local radiotherapy of the tumor, which effectively avoids systemic toxicity, achieves tumor regression on the tumor-bearing mouse model and inhibits unirradiated distal tumor growth (227).

Individual heterogeneity in immune background also presents a major challenge. Patients vary substantially in their baseline immune status, which consequently leads to differential responsiveness to TLR agonists (228). For instance, the drug HAP-ODN, a TLR9 agonist encapsulated by nanomaterials, exhibited significant variability in antitumor efficacy across different tumor-bearing mouse models and even among individuals within the same model. Prior to treatment, substantial differences were observed in multiple immune indicators between TLR9 responders and non-responders, including levels of inflammatory cytokines such as IL-12, IL-6, and TNF; the degree of intratumoral lymphocyte infiltration; and splenic condition (229). To address this challenge, it is essential to conduct in-depth investigation into patients’ immune characteristics, such as immune cell subtypes and cytokine secretion profiles, and to establish personalized immunotherapy models. Future clinical strategies should be more focused on biomarker-based patient selection, and more focused on the use of biomarkers for patient selection, technologies including single-cell sequencing can be employed to comprehensively analyze the TME and peripheral blood immune cells of patients, thereby identifying immune cell subpopulations sensitive to TLR agonists and related biomarkers. For example, high levels of dendritic cell (such as CD141 + cDC1) or specific cytokine signatures (such as high levels of IFN-γ) within the TME at baseline may predict better response to TLR agonists (230). These findings can serve as a basis for selecting suitable patients and developing individualized dosing regimens (231).

## 6 Conclusion

TIR domain-containing proteins serve as central signaling hubs bridging innate and adaptive immunity. Their role in tumor immunity is defined by a core characteristic of bidirectional regulation: they not only provide impetus for anti-tumor immunity but may also facilitate tumor immune escape. This complex regulatory mechanism not only underlies their unique value as therapeutic targets but also introduces both challenges and opportunities for clinical translation. This review systematically summarizes the central role and translational potential of TIR domain proteins in tumor immune regulation. However, it is crucial to recognize the persistent challenges in this field, including signaling complexity, dual-edged therapeutic effects, and individual variability. Future studies should extend beyond phenomenological descriptions to focus on deciphering signaling crosstalk networks, defining biomarkers, and developing intelligent delivery systems, ultimately enabling truly precise immunological interventions.

The core of the bidirectional role of TIR domain proteins lies in their regulation of the plasticity of the TME. In the context of anti-tumor immunity, TIR signaling initiates multi-tiered immune activation through both the MyD88 and TRIF pathways. The TLR4 agonist MPLA upregulates co-stimulatory molecules on DCs, enhances cross-presentation of tumor-associated antigens (TAAs), and promotes CD8^+^ T cell infiltration. Meanwhile, the TLR3 agonist Poly I:C activates the TRIF pathway to induce type I IFN secretion, stimulating a coordinated response between innate and adaptive immunity and improving the sensitivity to ICIs. Furthermore, TLR agonists can expand the repertoire of antitumor T cells through ICD, and synergistically activate a greater number of T cells when combined with ICIs.

However, aberrant activation of TIR signaling can also drive tumor progression. Within the TME, TLR4 recognizes HMGB1 to activate the MyD88/NF-κB pathway, enhancing breast cancer invasion and angiogenesis. In ovarian and colorectal cancers, sustained MyD88 activation promotes increased secretion of IL-10 and TGF-β, which suppresses effector T cell function and expands Tregs. Additionally, TIR signaling contributes to CSC stemness maintenance; for example, TLR3 cooperates with β-catenin and NF-κB to promote the transformation of breast cancer cells into CSCs, directly leading to therapy resistance. Given the bidirectional regulatory nature of TIR domain-containing proteins, future research should focus on the following directions to achieve precise immunomodulation.

First, combination therapy. The core of combination strategies lies in developing personalized regimens based on tumor type and TME characteristics. For instance, in immunologically cold tumors, Professor Hiroyoshi Nishikawa’s team (232) attempted to reverse the immunosuppressive state using the TLR agonist OK-432 in combination with a PD-1 blocker. OK-432 effectively activates antigen-presenting cells but also induces the accumulation of polymorphonuclear myeloid-derived suppressor cells (PMN-MDSCs) within the TME, which suppresses immune responses and contributes to therapy resistance. However, when combined with CXCR2-neutralizing antibodies or anti-Ly6G antibodies to reduce PMN-MDSC recruitment, this approach successfully reversed the immunosuppressive cold phenotype and enhanced the antitumor efficacy of the combination therapy (232). In diffuse large B-cell lymphoma (DLBCL) with the MyD88 L265P mutation caused by TIR domain mutation, studies have found that the histone deacetylase inhibitor panobinostat reduces the binding of STAT3 to the MyD88 promoter by inhibiting STAT3 phosphorylation, thereby downregulating MyD88 expression, and when combined with the BTK inhibitor ibrutinib, it more potently suppresses NF-κB activity and has been shown to induce tumor regression in DLBCL xenograft models (233).

Innovations in nanotechnology offer new approaches to address the delivery challenges in TIR-targeted therapy. Future nanocarriers must achieve dual functions: targeted accumulation and controllable release. First, modulating the physicochemical properties of nanoparticles (such as particle size and surface characteristics) to enhance the accumulation efficiency of TLR agonists within the TME. A research team developed a multifunctional nanoadjuvant (MPN/CpG) by integrating manganese ions (Mn²^+^) with the TLR9 agonist CpG. By optimizing its physicochemical structure, the MPN/CpG nanoadjuvant demonstrated significantly enhanced accumulation in lymph nodes and increased uptake by APCs within the TME, compared to free CpG (234). Second, developing responsive release systems to reduce systemic toxicity. For instance, a research team designed a nanotechnology-based R848 “bottlebrush prodrug” (BPDs) platform, in which the release kinetics of the TLR7/8 agonist R848 were precisely regulated by tuning the linker chemistry. After systemic administration, this system effectively accumulates at tumor sites and enables tumor-localized responsive release of R848. It not only enhances intratumoral immunostimulatory effects but also avoids immune-related toxicities associated with systemic administration, leading to effective tumor growth suppression and a favorable safety profile in mouse tumor models (235). Third, constructing co-delivery systems for multiple drugs to synergistically enhance therapeutic efficacy. A notable innovation is the Glypican-3-targeted macrophage-based formulation RILO@MG (236), which carries nanoparticles co-loaded with the TLR7/8 agonist R848 and the IDO1 inhibitor INCB024360. By leveraging a targeting peptide for specific binding to tumor cells, this system activates innate immunity via R848 while simultaneously blocking immunosuppressive pathways through INCB024360. This synergistic action potently enhances anti-tumor immune responses in tumor-bearing mouse models, demonstrating the therapeutic advantage of co-delivering TLR agonists with immune modulators.

In conclusion, as a crucial signaling hub in innate immunity and tumor immunity, TIR domain proteins have their bidirectional regulatory mechanisms providing a novel paradigm for tumor therapy. In the future, with precise targeting as the core, it is necessary to address their “double-edged sword” effect through stratified design of combination therapies, optimization of nano-delivery systems, assessment of individual immunity, and exploration of new functions. This will facilitate the transition from laboratory discoveries to clinical applications and open up a new avenue for precision in tumor immunotherapy.
